# Tumor Susceptibility Gene 101 (TSG101) Is a Novel Binding-Partner for the Class II Rab11-FIPs

**DOI:** 10.1371/journal.pone.0032030

**Published:** 2012-02-14

**Authors:** Conor P. Horgan, Sara R. Hanscom, Eoin E. Kelly, Mary W. McCaffrey

**Affiliations:** Molecular Cell Biology Laboratory, Department of Biochemistry, BioSciences Institute, University College Cork, Cork, Ireland; Institut Européen de Chimie et Biologie, France

## Abstract

The Rab11-FIPs (Rab11-family interacting proteins; henceforth, FIPs) are a family of Rab11a/Rab11b/Rab25 GTPase effector proteins implicated in an assortment of intracellular trafficking processes. Through proteomic screening, we have identified TSG101 (tumor susceptibility gene 101), a component of the ESCRT-I (endosomal sorting complex required for transport) complex, as a novel FIP4-binding protein, which we find can also bind FIP3. We show that α-helical coiled-coil regions of both TSG101 and FIP4 mediate the interaction with the cognate protein, and that point mutations in the coiled-coil regions of both TSG101 and FIP4 abrogate the interaction. We find that expression of TSG101 and FIP4 mutants cause cytokinesis defects, but that the TSG101-FIP4 interaction is not required for localisation of TSG101 to the midbody/Flemming body during abscission. Together, these data suggest functional overlap between Rab11-controlled processes and components of the ESCRT pathway.

## Introduction

Animal cytokinesis is a fundamental cellular process in which a dividing cell partitions its contents leading to the formation of two diploid daughter cells. A key requirement of cytokinesis is the spatial and temporal assembly and activation of an acto-myosin contractile-ring at the equatorial cortex of the cell [1], [2]. Constriction of this contractile-ring results in the formation of a circumferential depression in the plasma membrane, known as the cleavage furrow. Upon further ingression of this furrow, in a process requiring additional constriction of the contractile-ring as well as insertion of new membrane into the cleavage furrow, a membrane-bound intercellular bridge known as the midbody is formed. Cytokinesis is completed by severance of the midbody in a process known as abscission. During the past decade, a significant amount of information has emerged which implicates disparate endosomal protein machinery in the processes of abscission (reviewed in [3]). Prominent among these reports are components of the ESCRT (endosomal sorting complex required for transport) complexes, as well as members of the Rab GTPase family.

ESCRTs (ESCRT-0, ESCRT-I, ESCRT-II and ESCRT-III) are multi-component protein complexes that are conserved from archaea to animals and are implicated in the trafficking of endosomal cargo destined for lysosomal degradation as well as multivesicular body (MVB) biogenesis [4], [5]. They recognise ubiquitinated receptors and facilitate their sorting into endosomal membrane invaginations by deforming the membrane during inward vesiculation of MVBs [6]. ESCRTs then function in the scission events that result in generation of the intraluminal vesicles (ILVs) within MVBs [6]. ESCRTs have also been implicated in further cellular processes requiring membrane scission events; namely, the budding of enveloped viruses and closure of the intercellular bridge during cytokinetic abscission [7]–[10]. In this respect, one of the four proteins that constitute the ESCRT-I complex, TSG101 (tumor susceptibility gene 101) (Vps23 in yeast), is recruited to the Flemming body during cytokinesis and is required for successful completion of abscission [11]–[15]. The ESCRT-III complex is also necessary for abscission [14], [16]–[18].

In humans, Rab GTPases are a family of over 60 proteins that act as key regulators of all stages of intracellular membrane trafficking [19]. They act as molecular switches by alternating between active and inactive conformations which are dependent upon the nucleotide-bound state of the Rab [19]. When GTP-bound, Rab proteins are active and execute precise trafficking steps through the recruitment of downstream effector proteins [19], [20]. Members of the Rab11-subfamily (Rab11a, Rab11b and Rab25) are distributed to endosomal membranes, and among their identified effectors are a conserved protein family termed the Rab11-FIPs (Rab11-family interacting proteins; henceforth, FIPs), which bind Rab11 via a carboxy-terminal Rab11-binding domain (RBD) [21]. The class I FIPs (RCP, Rip11 and FIP2) contain C2-domains at their amino-termini and have been implicated in the recycling of a variety of endocytic cargoes [21]. Conversely, the class II FIPs have amino-terminal EF-hand motifs and FIP3 has an extensive amino-terminal proline-rich region (PRR) [21]. FIP3, in conjunction with Rab11, is involved in endosomal-recycling processes [22], [23]; and FIP4 plays a role in the regulation of retinal development in zebrafish (*Danio rerio*) [24], [25]. Additionally, both class II FIPs have been implicated in the abscission step of cytokinesis [3], [21], [26]–[29].

While FIP4 has been implicated in retinal development and cytokinesis, it is perhaps the FIP for which the least data exists in the literature. Here, we have embarked on a yeast two-hybrid proteomic screen to identify novel FIP4-binding proteins and through this, and subsequent experiments, have identified the ESCRT-I component TSG101 as a novel binding-partner for both of the class II FIPs.

## Results

### TSG101 is a novel binding-partner for the class II FIPs

**Figure 1:**
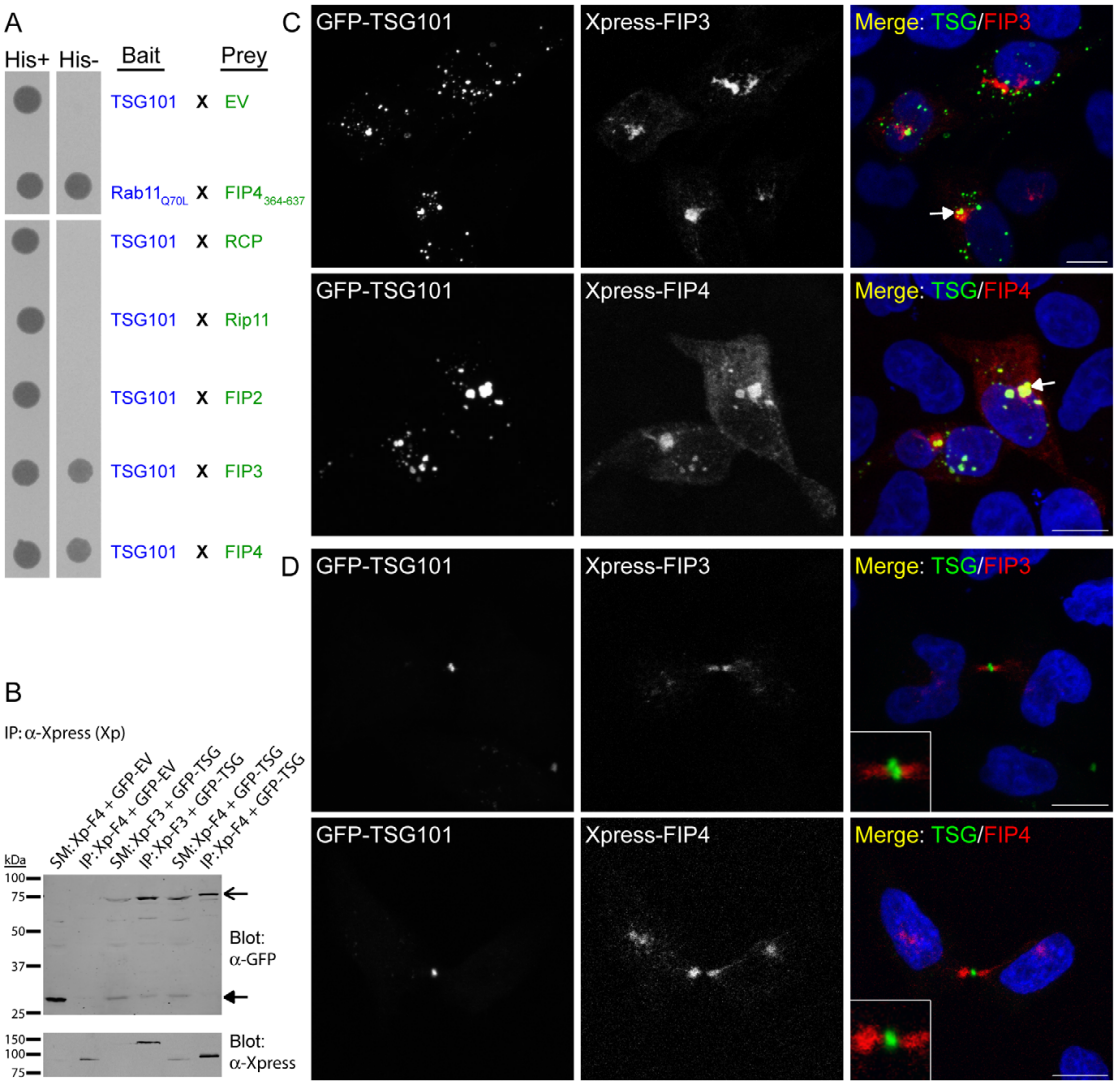
TSG101 binds the class II FIPs. (***A***) Yeast two-hybrid analysis of the interaction between the indicated proteins. Protein-protein interactions were determined by the ability of the transformed yeast to grow on minimal medium lacking tryptophan, leucine and histidine (*His−*). *EV*, empty vector. (***B***) Co-immunoprecipitation analysis of the ability of Xpress-FIPs to co-immunoprecipitate GFP-TSG101 in HeLa cells using an anti-Xpress antibody (*SM*, starting material; *IP*, immunoprecipitate). Co-immunoprecipitated proteins were revealed using an anti-GFP antibody. GFP-empty vector (*EV*) was used as a control. SM load was 3.33%. (***C*** and ***D***) HeLa cells were transfected with constructs encoding the indicated proteins. At 16–18 hours post-transfection, cells were processed for immunofluorescence microscopy and immunostained with an anti-Xpress antibody. Cells expressing relatively low levels of the GFP-TSG101 protein are shown in *D*. DAPI was used to visualise the nuclei. Images were acquired by confocal microscopy. Insets illustrate the midbody region of dividing cells at 2.5× higher magnification. Scale bar indicates 10 µm. Data are typical of at least three independent experiments.

To identify novel FIP4-interacting proteins, full-length FIP4 was used as bait to screen an adult human brain cDNA library using the yeast two-hybrid system. Forty resultant clones were sequenced; of which 18 corresponded to FIP4 itself, and five corresponded to FIP3. Given that the class II FIPs are known to dimerise in the yeast two-hybrid system [30], [31], these data indicate that the screen was successful. Of the remaining 17 clones, seven were determined to be TSG101. To determine if further members of the FIP family could also bind TSG101, we tested the ability of each of the FIPs to bind TSG101 in the yeast two-hybrid system. For these experiments, L40 *Saccharomyces cerevisiae* were co-transformed with constructs encoding TSG101 and each of the FIPs, and assayed for the ability of transformed yeast to grow on selective medium lacking histidine. We found that while TSG101 displayed no binding to the class I FIPs, it interacted with both FIP3 and FIP4 (Figure 1A). Biochemical experiments in HeLa cells confirmed this result as Xpress-fused FIP3 and FIP4 could co-immunoprecipitate GFP-fused TSG101 (Figure 1B). Next, we examined the distribution of the class II FIPs with respect to that of TSG101 in HeLa cells by confocal microscopy. Previous studies have demonstrated that exogenously-expressed class II FIPs predominantly localise to the Rab11-positive endosomal-recycling compartment (ERC), and that their overexpression compacts this compartment, as well as many class II FIP-binding proteins, into a pericentrosomal location [22], [23], [26], [30], [32]. In interphase HeLa cells, we found that when Xpress-FIP3 or FIP4 were co-expressed with GFP-TSG101, the FIP proteins were predominantly present in the perinuclear region of the cell, while the TSG101 was found in punctate structures dispersed throughout the cell (Figure 1C). The degree of co-localisation observed between the class II FIPs and TSG101 varied widely between cells; approximately 37% of cells co-expressing Xpress-FIP3 and GFP-TSG101 and 32% of cells co-expressing Xpress-FIP4 and GFP-TSG101 displayed little or no co-localisation; approximately 46% (FIP3/TSG101) and 47% (FIP4/TSG101) had limited, albeit some, co-localisation; and approximately 17% (FIP3/TSG101) and 21% (FIP4/TSG101) displayed strong co-localisation which was usually most evident in cells expressing relatively high levels of both proteins (Figure 1C; arrow in lower panel). As the class II FIPs and TSG101 have previously been implicated in cytokinesis, we also examined the distribution of the class II FIPs with respect to TSG101 in cells undergoing the terminal stages of cell division. Consistent with previous studies [26]–[29], [33], we found that during cytokinesis, the class II FIPs localised within the midbody, the membrane-bounded intercellular canal between the dividing cell (Figure 1D). As expected [11], [13], GFP-TSG101 was also found within the midbody, but unlike the class II FIPs, it was predominantly present on the Flemming body, the electron-dense centre of the midbody (also known as the midbody-ring) (Figure 1D). While both sets of proteins were present within the midbody in cells undergoing abscission/cytokinesis, little co-localisation was observed between either of the class II FIPs and TSG101 (Figure 1D, insets).

### α-helical coiled-coil regions in both TSG101 and FIP4 mediate the interaction between the two proteins

**Figure 2:**
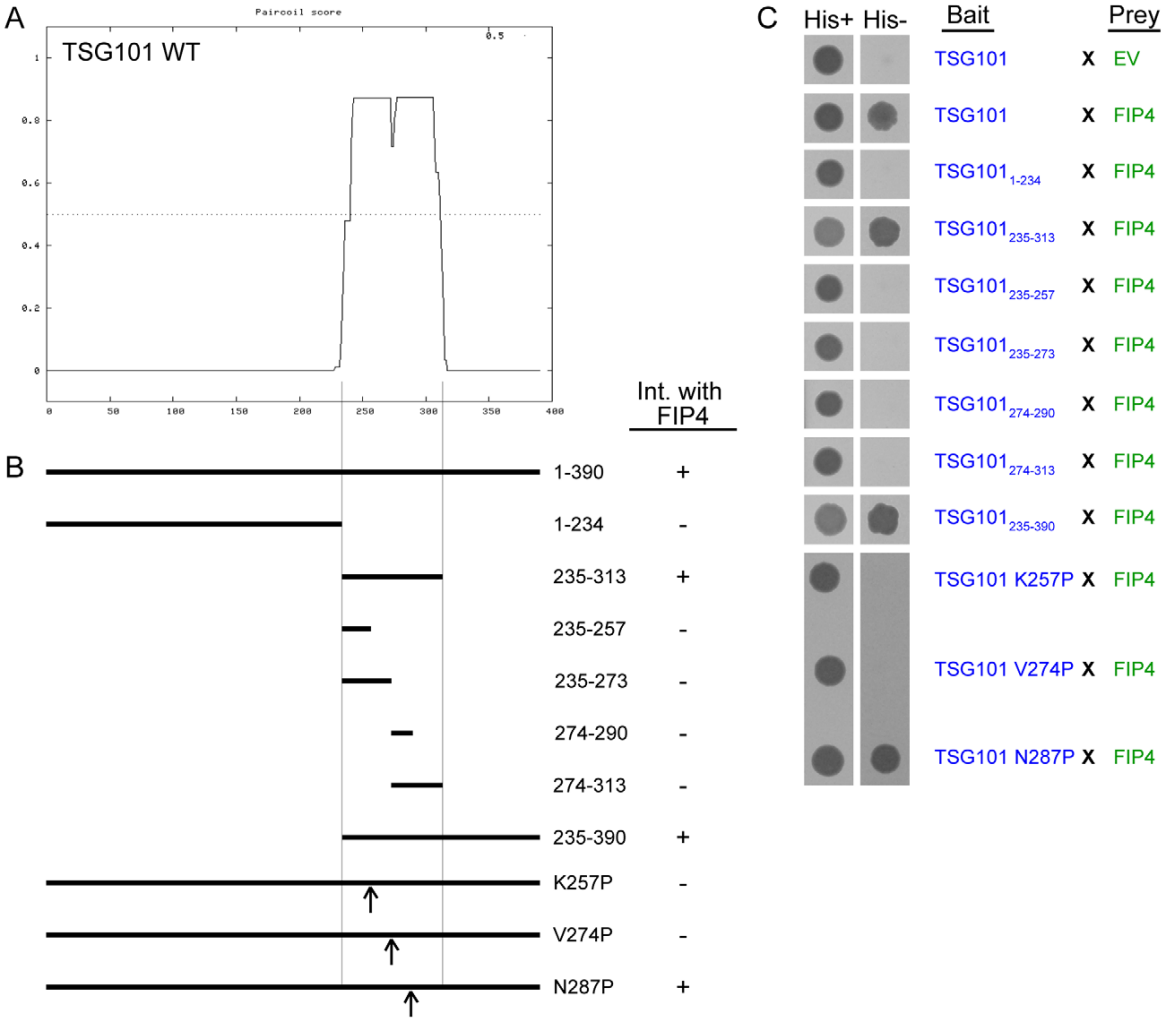
The coiled-coil region of TSG101 mediates the interaction with FIP4. (***A***) Plot depicting the probability of α-helical coiled-coil structure formation in TSG101 as determined using the *PairCoil* algorithm. (***B***) Schematic representation of the TSG101 truncation and point mutants that were tested for FIP4-binding ability. The outcome of the yeast two-hybrid experiments performed (part *C*) are indicated adjacent to the corresponding mutant in the schematic. (***C***) Yeast two-hybrid analysis of the interaction between the indicated proteins. Protein-protein interactions were determined by the ability of the transformed yeast to grow on minimal medium lacking tryptophan, leucine and histidine (*His−*). *EV*, empty vector. Data are typical of at least three independent experiments.

**Figure 3:**
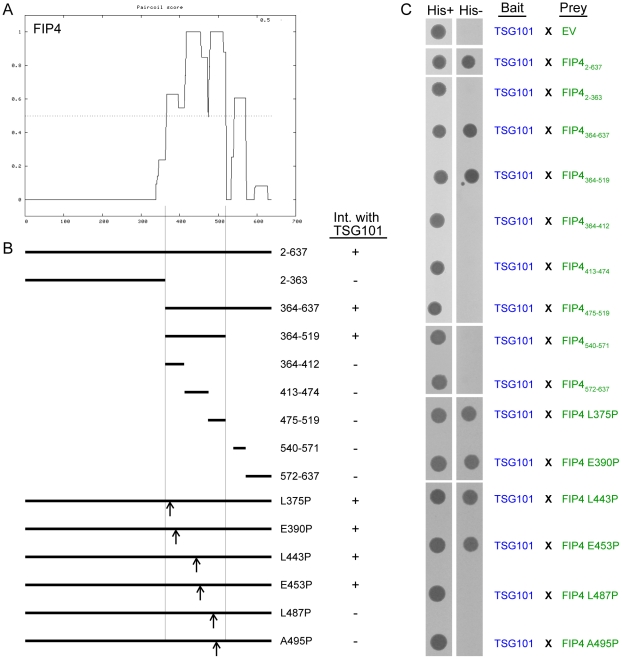
An extensive coiled-coil region of FIP4 mediates the interaction with TSG101. (***A***) Plot depicting the probability of α-helical coiled-coil structure formation in FIP4 as determined using the *PairCoil* algorithm. (***B***) Schematic representation of the FIP4 truncation and point mutants that were tested for TSG101-binding ability. The outcome of the yeast two-hybrid experiments performed (part *C*) are indicated adjacent to the relevant mutant in the schematic. (***C***) Yeast two-hybrid analysis of the interaction between the indicated proteins. Protein-protein interactions were determined by the ability of the transformed yeast to grow on minimal medium lacking tryptophan, leucine and histidine (*His−*). *EV*, empty vector. Data are typical of at least three independent experiments.

To further explore the significance of the class II FIP-TSG101 associations, we concentrated our efforts on the TSG101/FIP4 interaction. In order to generate TSG101 and FIP4 mutants that should act as dominant-negative mutants with respect to the cognate protein, we mapped the regions of the TSG101 and FIP4 proteins that mediate this interaction. For this work, an extensive range of TSG101 and FIP4 truncation mutants were generated, and their ability to bind the cognate protein tested in the yeast two-hybrid system (Figure 2 and Figure 3). We narrowed down the minimal FIP4-binding region of TSG101 to amino acid residues 235–313, which corresponds to the α-helical coiled-coil domain present in TSG101 (Figure 2). Further truncation of this α-helical coiled-coil domain disrupted the interaction (Figure 2). Similarly, we found that an α-helical coiled-coil region of FIP4, amino acids 364–519, mediated the interaction with TSG101, and that further truncation of this region also blocked the interaction (Figure 3). Next, we sought to create full-length TSG101 and FIP4 proteins that had point mutations rendering them unable to bind the cognate protein. To this end, we utilised the *Paircoil* algorithm [34] to predict the effect of a proline substitution for each of the amino acids within the α-helical coiled-coil domains of TSG101 and FIP4 on the probability of α-helical coiled coil formation. From these predictions, three TSG101 point mutants (K257P, V274P and N287P) and six FIP4 point mutants (L375P, E390P, L443P, E453P, L487P and A495P) were identified as being likely to perturb TSG101 and FIP4 α-helical coiled-coil formation (Figure S1 and Figure S2). We then tested the ability of each of these mutants to bind the cognate protein in the yeast two-hybrid system and found that two of the three TSG101 point mutants (K257P and V274P) abrogated the interaction with FIP4 (Figure 2), and two of the six FIP4 point mutants (L487P and A495P) blocked the interaction with TSG101 (Figure 3).

### Expression of TSG101 and FIP4 dominant-negative mutants result in cytokinesis defects

**Figure 4:**
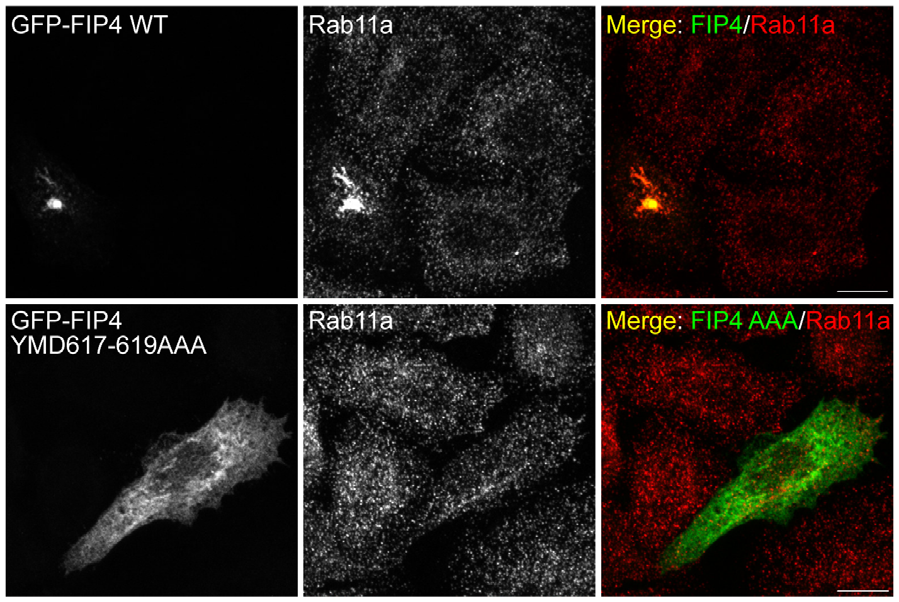
FIP4 YMD617–619AAA is deficient in Rab11-binding. HeLa cells were transfected with constructs encoding the indicated proteins. At 16–18 hours post-transfection, cells were processed for immunofluorescence microscopy and immunostained with an anti-Rab11a antibody. Images were acquired by confocal microscopy. Scale bar indicates 10 µm. Data are typical of at least three independent experiments.

As TSG101 and FIP4 have been previously implicated in cytokinesis [11], [13], [27], [28], we investigated the effect of expression of the aforementioned TSG101 and FIP4 truncation and point mutants on the ability of cells to successfully complete cytokinesis. In addition, as FIP4 is a Rab11 effector protein, we also generated a FIP4 mutant that was deficient in Rab11a-binding and tested its ability to prevent successful cytokinesis. Previous studies have shown that point mutations in the conserved YID/YMD motif within the RBD of the FIPs blocks the interaction with Rab11a [32], [35] (also see Figure S3A). Therefore, constructs encoding GFP-fusions of FIP4 that had point mutations in this YID/YMD motif (M618E and D619A) were generated, and their ability to co-localise with endogenous Rab11a in HeLa cells assessed by confocal microscopy. We found that, relative to the wild-type protein, the FIP4 M618E and D619A mutants were considerably distributed to the cytosol, and displayed reduced co-localisation with Rab11a (Figure S3B). Nevertheless, in some cells, these mutants retained co-localisation with Rab11a, indicative that they retained some, albeit likely reduced, ability to bind Rab11a (Figure S3B). Therefore, we created a FIP4 mutant in which we substituted the FIP4 YID/YMD motif with three alanine residues (YMD617–619AAA). When we examined the cellular distribution of the full-length GFP-FIP4 YMD617–619AAA mutant, we found that, unlike the wild-type GFP-FIP4 protein which strongly co-localises with Rab11a and condenses the Rab11a-positive compartment into the pericentrosomal region, the YMD617–619AAA mutant was predominantly cytosolic and exhibited virtually no co-localisation with Rab11a (Figure 4). Together, these data indicate that the FIP4 YMD617–619AAA is deficient in Rab11a-binding and should serve as a dominant-negative mutant with respect to Rab11a-mediated FIP4 cellular function.

**Figure 5:**
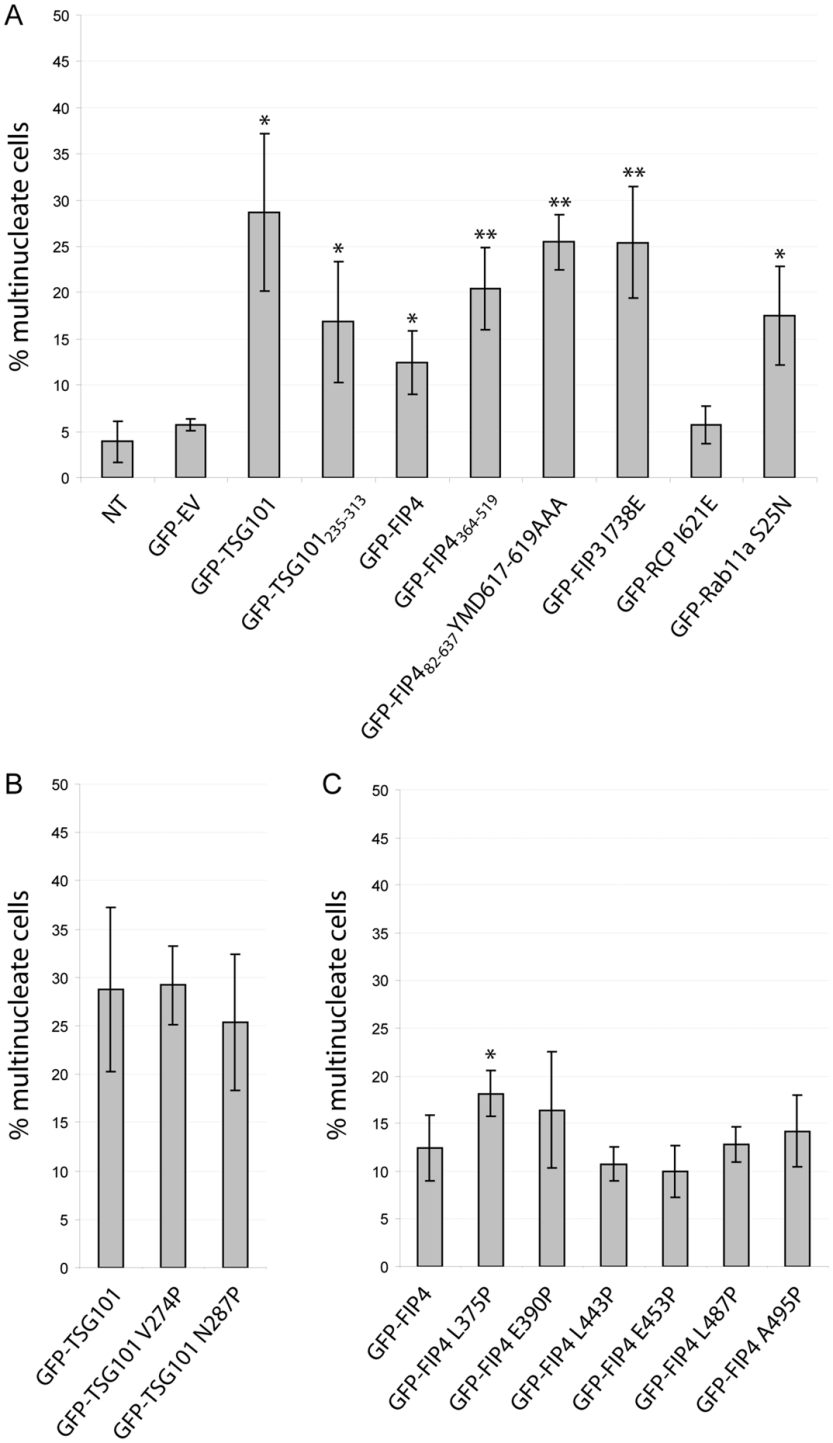
Expression of TSG101 and FIP4 dominant-negative mutants cause abscission failure. HeLa cells were transfected with constructs encoding the indicated proteins. At 36–40 hours post-transfection, cells were processed for immunofluorescence microscopy, immunostained for α-tubulin and their nuclei fluorescently-labelled with DAPI. A minimum of 150 transfected cells per experiment were counted and scored for multinucleation (>1 nucleus). Results, from three independent experiments, are expressed as the mean percentages ± S.D. Statistical significance was determined using an unpaired *t* test to investigate: (***A***) the difference between empty vector and GFP-fusion means, (***B***) the difference between GFP-TSG101 and GFP-TSG101 point mutant means and (***C***) the difference between GFP-FIP4 and GFP-FIP4 point mutant means. Statistical significance, *p<0.05, **p<0.02. *NT*, non-transfected; *EV*, empty vector.

To examine the effect of expression of our TSG101 and FIP4 mutants on the ability of cells to successfully complete cytokinesis, HeLa cells were transfected with GFP-fusions of the wild-type or mutant TSG101 or FIP4 proteins for 36–40 hours, fixed, immunostained for α-tubulin and their nuclei fluorescently-labelled with DAPI, and then scored for multinucleation (two or more nuclei). We found that in HeLa cells expressing GFP-TSG101, approximately 29% of cells were multinucleated, and approximately 17% of cells expressing GFP-TSG101_235–313_ (the FIP4-binding region) had more than one nucleus (compare with an approximate 6% multinucleation rate in GFP-empty vector-expressing cells) (Figure 5A). Multinucleation in GFP-TSG101 and GFP-TSG101_235–313_-expressing cells correlated with high levels of expression of the exogenous polypeptides (data not shown). Expression of the GFP-FIP4 wild-type protein also caused multinucleation (12%), and expression of GFP-FIP4_364–519_ (the TSG101-binding region) had a more profound effect on the ability of cells to successfully complete cytokinesis, as approximately 20% were multinucleate (Figure 5A). We also found that expression of the FIP3 and FIP4 mutants that are deficient in Rab11a-binding (FIP3 I738E and FIP4 YMD617–619AAA) strongly inhibited cytokinesis as approximately 25% of cells displayed a multinucleate phenotype; whereas expression of RCP I621E, an equivalent mutant of a class I FIP, failed to result in multinucleation levels above that of controls (Figure 5A). Consistent with a previous study [28], we also found that expression of the dominant-negative Rab11a mutant (Rab11a S25N) caused cytokinesis failure as 17.5% of cells were multinucleate (Figure 5A).

We also examined the effects of expression of the TSG101 and FIP4 proteins with the single amino acid substitutions in their α-helical coiled coil domain that, in some cases, disrupt the interaction with the cognate protein, on the ability of cells to successfully complete cytokinesis. We found no significant difference in the proportion of cells displaying a multinucleation phenotype between GFP-TSG101 wild-type, GFP-TSG101 V274P (FIP4-binding deficient mutant) or GFP-TSG101 N287P (FIP4-binding unaffected) (Figure 5B). In addition, we found that expression of the TSG101-binding deficient mutants of FIP4 (L487P and A495P) failed to result in multinucleation levels significantly above that of the GFP-FIP4 wild-type protein (Figure 5C). Notably, expression of FIP4 L375P, a FIP4 mutant that retains its TSG101-binding ability, did result in multinucleation levels above that of GFP-FIP4 wild-type (Figure 5C).

### TSG101 localises to the Flemming body independently of the class II FIPs

**Figure 6:**
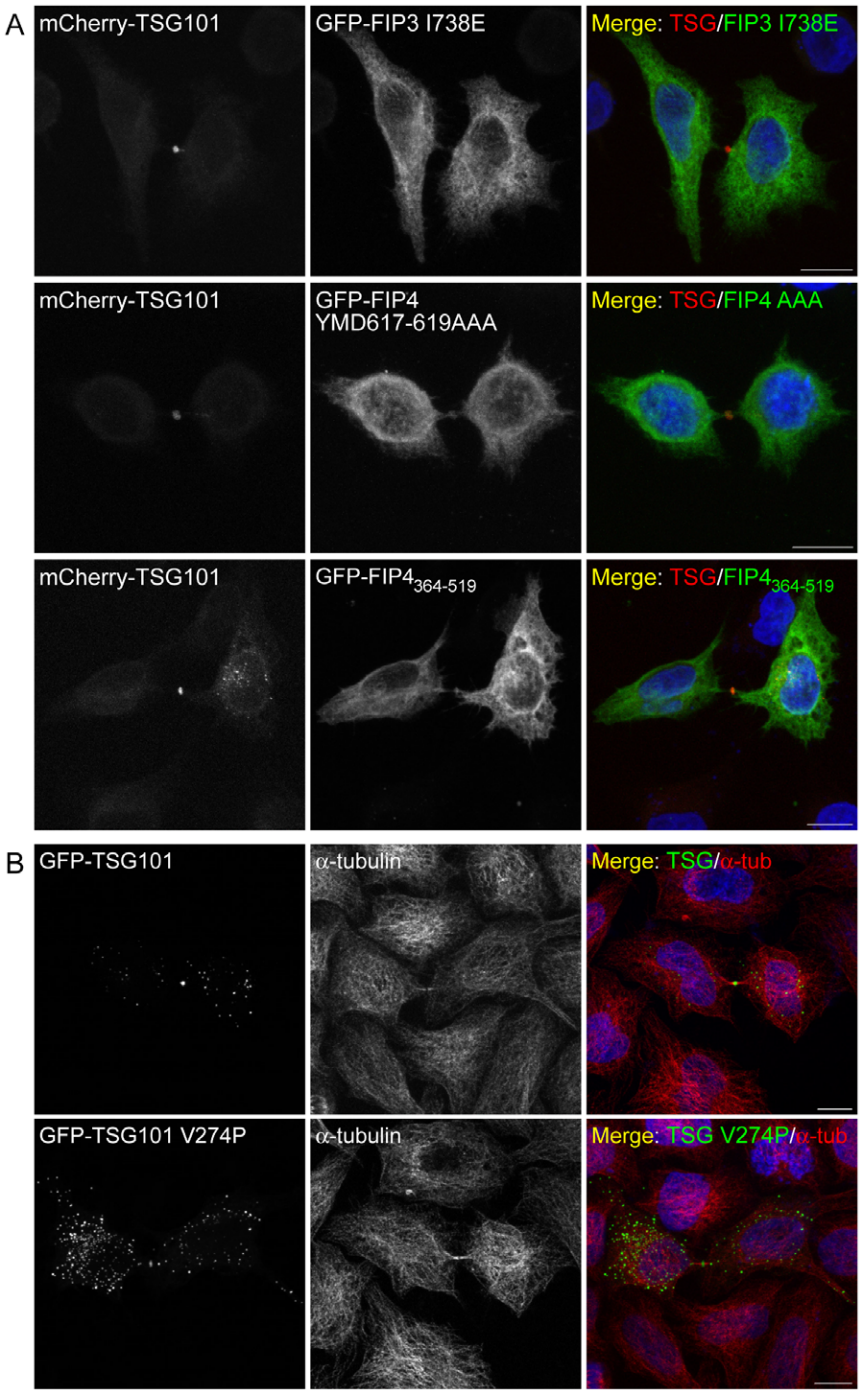
TSG101 localises to the Flemming body during abscission independently of the class II FIPs. (***A*** and ***B***) HeLa cells were transfected with constructs encoding the indicated proteins. At 16–18 hours post-transfection, cells were processed for immunofluorescence microscopy and, where indicated, immunostained for α-tubulin. DAPI was used to visualise the nuclei. Images, from cells expressing relatively low levels of the TSG101 fusion protein, were acquired by confocal microscopy. Scale bar indicates 10 µm. Data are typical of at least three independent experiments.

**Figure 7:**
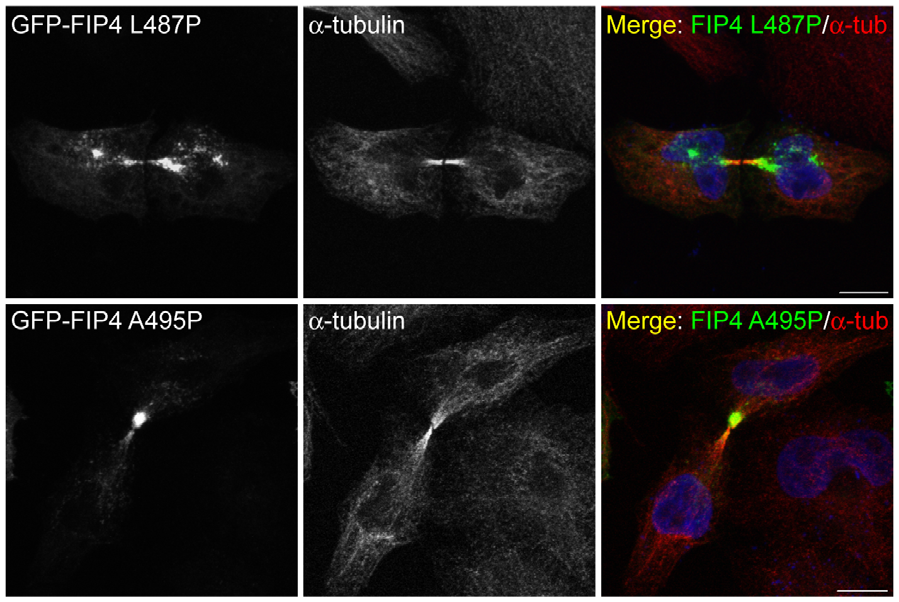
FIP4 localises to the midbody of dividing cells independently of TSG101. HeLa cells were transfected with constructs encoding the indicated proteins. At 16–18 hours post-transfection, cells were processed for immunofluorescence microscopy and immunostained for α-tubulin. DAPI was used to visualise the nuclei. Images were acquired by confocal microscopy. Scale bar indicates 10 µm. Data are typical of at least three independent experiments.

Previous studies have demonstrated that CEP55 (centrosome protein 55), a centrosome and midbody protein involved in abscission, is required for recruitment of TSG101 to the midbody during cytokinesis [11], [13]. To ascertain if the class II FIPs are also required for localisation of TSG101 to the midbody during cytokinesis, we investigated the ability of mCherry-TSG101 to localise to the Flemming body in cells expressing class II FIP dominant-negative mutants. We found that in HeLa cells co-expressing mCherry-TSG101 together with GFP-fusions of FIP3 I738E, FIP4 YMD617–619AAA or FIP4_364–519_, that localisation of the TSG101 protein to the Flemming body was not impeded (Figure 6A). In addition, we found that like the wild-type protein, the FIP4-binding deficient TSG101 mutant (TSG101 V274P) localises to the Flemming body during cytokinesis (Figure 6B). We also determined if interaction between TSG101 and FIP4 was required for localisation of FIP4 to the midbody and found that the TSG101-binding deficient mutants of FIP4 (FIP4 L487P and FIP4 A495P) were not precluded from localisation to the midbody during cytokinesis (Figure 7).

## Discussion

During the past decade, a multitude of endosomal proteins have been implicated in animal cytokinesis which underscores the crucial importance of intracellular trafficking processes in the completion of cell division [3]. In this regard, recent evidence suggests that ESCRT-I and ESCRT-III components are sequentially recruited to the central region of the intercellular bridge where they lead to membrane deformation, and ultimately, breakage of the midbody during abscission [15], [17], [18]. To identify novel proteins implicated in Rab11-controlled cellular processes, we performed a proteomic screen with FIP4 as bait, and have identified TSG101 as a novel FIP4-binding protein. Upon investigation of the extent of FIP-TSG101 interactions, we found that TSG101 also binds FIP3, the other class II FIP. We also found that the α-helical coiled-coil domains of FIP4 and TSG101 are sufficient for binding of the cognate protein, and that point mutations in either of these α-helical coiled-coil domains blocks this interaction.

Expression of GFP-fused wild-type TSG101 or FIP4 were found to result in multinucleation in HeLa cells. These data are consistent with previous reports implicating TSG101 and the class II FIPs in cytokinesis [12], [13], [15], [26]–[28], [36], and indicate that these proteins may form functional complexes with each other during the terminal stages of cell division. Nevertheless, when we examined the distribution of FIP3 and FIP4 with respect to that of TSG101 in cytokinetic cells, we failed to detect significant midbody co-localisation between either protein pair, despite the presence of each of these proteins within the midbody. In addition, while expression of TSG101 and class II FIP truncation mutants did cause multinucleation, expression of TSG101 and FIP4 point mutants deficient in binding the cognate protein, while also causing multinucleation, failed to result in multinucleation levels significantly above that of the wild-type proteins. We also found that localisation of TSG101 to the Flemming body was not impeded in cells expressing dominant-negative Rab11a-binding deficient mutants of FIP3 or FIP4; and furthermore, the TSG101 V274P mutant, which cannot bind FIP4, can also localise to the Flemming body. Together, these data indicate that while TSG101 and the class II FIPs are clearly necessary for successful completion of cytokinesis, the class II FIPs are not required for the trafficking of TSG101 to the midbody during telophase/cytokinesis and that TSG101 and the class II FIPs may not form functional complexes during cytokinesis. This raises the possibility that a functional interaction may exist between TSG101 and the class II FIPs in distinct ESCRT-mediated cellular events. In this regard, while it is possible that the class II FIPs could play a role in cargo sorting or MVB biogenesis, to our knowledge, no data exists implicating either FIP3 or FIP4 in the endocytic degradative pathway. Interestingly, the ESCRT complexes are implicated in the budding of enveloped viruses [9], [10], and recent reports indicate that Rab11 is involved in influenza A virus budding and filament formation [37]. FIP3 was also found to be required for formation of influenza A viral filaments [37]. Furthermore, FIP4 was recently found to bind the human cytomegalovirus (HCMV) envelope glycoprotein M (UL100), and expression of the Rab11 S25N (dominant-negative mutant) and depletion of FIP4 expression in HCMV-infected cells, led to a decrease in infectious virus production [38]. These studies point to roles for Rab11 and the class II FIPs in the cellular events leading to viral envelopment which may involve class II FIP/ESCRT interactions.

In summary, we have identified a component of the ESCRT-I complex as a novel binding-partner for a subset of Rab11 effectors. The major challenge now remains to elucidate the functional links between Rab11, its effectors, and the ESCRT complexes; and to determine how further Rab GTPases may influence intracellular trafficking events along the ESCRT-pathway.

## Materials and Methods

### Yeast two-hybrid screen and assay

The yeast two-hybrid screen with full-length FIP4 as bait was screened against an adult human brain cDNA library [ProQuest human brain cDNA library (11376-027) (Invitrogen)] by Creative Biolabs (Shirley, New York, USA). For the yeast two-hybrid assay, constructs encoding the polypeptides of interest in pVJL10 (bait) and pGADGH (prey) two-hybrid vectors were co-transformed into the *S. cerevisiae* L40 reporter strain using the following procedure. A YPD-agar plate was streaked with *S. cerevisiae* and incubated at 30°C until sufficient colonies had formed (3–5 days). 10 ml of YPD media was inoculated with a colony from the YPD-agar plate and incubated overnight at 30°C with rotation at 225 rpm. The *S. cerevisiae*/YPD culture was diluted 1∶10 with fresh YPD media and incubated for 2 hours at 30°C with rotation at 225 rpm (cell density between 10^7^ and 3.0×10^7^ cells per ml). The cells were pelleted by centrifugation at 580× *g*, and the pellet washed with 90 ml of 0.1 M LiAc/TE. The cells were again pelleted, resuspended in 2 ml of 0.1 M LiAc/TE, and incubated for 1 h at 30°C with rotation at 225 rpm. For each reaction, 150 µl of the yeast/LiAc/TE solution was added to Eppendorf tubes containing 2 µg of each of the bait and prey plasmids, and 40 µg of heat-denatured salmon sperm DNA (Sigma), and the samples incubated for 10 min at 30°C. 500 µl of 50% PEG/LiAc/TE was added to each tube and the samples mixed by gentle inversion. Samples were incubated for 1 h at 30°C, with occasional mixing and thermally-shocked for 25 min in a water bath at 42°C, and the cells pelleted by centrifugation at 300× *g* for 5 s. The cells were washed twice by resuspension in 1 ml of YPD media and pelleted by centrifugation for 5 s at 300× *g*. The cells were resuspended in 100 µl of YPD media, plated onto agar plates containing selective media lacking tryptophan and leucine (W^−^L^−^) and incubated for 2–3 days at 30°C. Colonies from each plate were resuspended in an Eppendorf tube containing 500 µl of dH_2_O; and 5 µl of this solution spotted onto W^−^L^−^ agar plates [indicated as *His^+^* in the figures] or agar plates containing selective media lacking tryptophan, leucine and histidine [(W^−^L^−^H^−^); indicated as *His^−^* in the figures] and containing 0, 1, 5 or 10 mM 3-AT (3-Amino-1,2,4-triazole), an inhibitor of auto-activation, and incubated for 2–3 days at 30°C. The resultant spots were imaged using a FUJIFILM FinePix S602 Zoom digital camera. In all instances, images from the 1 mM 3-AT-containing W^−^L^−^H^−^ agar plates are shown.

### Cell line, primary antibodies and plasmid transfection

The HeLa (human cervical carcinoma) cell line, which was obtained from the European Molecular Biology Laboratory (EMBL) [39], was cultured in Dulbecco's modified Eagle's medium supplemented with 10% (v/v) foetal bovine serum, 2 mM L-glutamine and 25 mM HEPES and grown in 5% CO_2_ at 37°C. The mouse monoclonal antibodies used were anti-Xpress (Invitrogen) and anti-α-tubulin (Sigma). Rabbit polyclonal antibodies used were anti-GFP (Abcam) and anti-Rab11a (Zymed). Cells were transfected with plasmid constructs using TurboFect (Fermentas) as transfection reagent.

### Plasmid construction

The following plasmids have been previously described, subcloned, generated by site-directed mutagenesis (SDM) or PCR using the indicated primers. pEGFP-C1/FIP4 [22]; pEGFP-C1/FIP4_2–363_ (SDM: *Fwd* gacagcctgaccaatggggactagaagagcaagctgaagcaagag, *Rev* ctcttgcttcagcttgctcttctagtccccattggtcaggctgtc); pEGFP-C3/FIP4_364–519_ (subcloned from pGADGH/FIP4_364–519_); pEGFP-C3/FIP4_364–637_ (subcloned from pGADGH/FIP4_364–637)_; pEGFP-C3/FIP4_520–637_ (PCR: *Fwd* cccggatccaccaggcaggggccgcagtgcct, *Rev* cccgaattcttagtgtttgatctcgaggatggag); pEGFP-C1/FIP4 L375P (SDM: *Fwd* agagaacacacagccggtgcacagggtgc, *Rev* gcaccctgtgcaccggctgtgtgttctct); pEGFP-C1/FIP4 E390P (SDM: *Fwd* tggtgaaggatcagccgaccacggccgagc, *Rev* gctcggccgtggtcggctgatccttcacca); pEGFP-C1/FIP4 L443P (SDM: *Fwd* aacaacagtgactcggcccaagtctcaaacagaga, *Rev* tctctgtttgagacttgggccgagtcactgttgtt); pEGFP-C1/FIP4 E453P (SDM: *Fwd* gagaaactggatgagccgcggcagcgcatgtc, *Rev* gacatgcgctgccgcggctcatccagtttctc); pEGFP-C1/FIP4 L487P (SDM: *Fwd* gcgacagaaccgccctgagttccagaagg, *Rev* ccttctggaactcagggcggttctgtcgc); pEGFP-C1/FIP4 A495P (SDM: *Fwd* aggagcgggagccgacgcaggag, *Rev* ctcctgcgtcggctcccgctcct); pEGFP-C1/FIP4_82–637_ (subcloned from pGADGH/FIP4_82–637_ [30]); pEGFP-C1/FIP4_82–637_ YMD617–619AAA (SDM: template pEGFP-C1/-FIP4_82–637_ D619A, *Fwd* caacttccggctgaggcaggccgcggccaagattatcctcgcc, *Rev* ggcgaggataatcttggccgcggcctgcctcagccggaagttg); pEGFP-C1/FIP4 YMD617–619AAA (FIP4_2–344_ subcloned from pEGFP-C1/FIP4 into pEGFP-C1/FIP4_82–637_ YMD617–619AAA); pEGFP-C1/-FIP4_82–637_ M618E (SDM: *Fwd* cggctgaggcagtacgaggacaagattatcctcgc, *Rev* gcgaggataatcttgtcctcgtactgcctcagccg); pEGFP-C1/-FIP4_82–637_ D619A (SDM: *Fwd* aggcagtacatggccaagattatcctc, *Rev* gaggataatcttggccatgtactgcct); pEGFP-C3/Rab11a S25N (subcloned from pGEM/Rab11a S25N [40]); pEGFP-C3/RCP I621E [35]; pEGFP-C1/FIP3 I738E [41]; pCR3.1-GFP/TSG101 [11]; pGEX-3X/TSG101 (PCR: template pCR3.1-GFP/TSG101, *Fwd* cccgaattctatggcggtgtcggagagccagc, *Rev* cccgaattcctagtagaggtcactgagaccgg); pEGFP-C1/TSG101 (subcloned from pGEX-3X/TSG101); pmCherry-C1/TSG101 (subcloned from pGEX-3X/TSG101); pEGFP-C2/TSG101_235–313_ (subcloned from pVJL10-TSG101_235–313_); pEGFP-C2/TSG101_235–390_ (subcloned from pVJL10-TSG101_235–390_); pEGFP-C2/TSG101 V274P (subcloned from pVJL10-TSG101 V274P); pEGFP-C1/TSG101 N287P (SDM: *Fwd* tcaagaagtagccgaggttgataaacccatagaacttttgaaaaagaagga, *Rev* tccttctttttcaaaagttctatgggtttatcaacctcggctacttcttga); pEGFP-C1, pEGFP-C2 and pEGFP-C3 (Clontech); pcDNA3.1-HisB/FIP3 (Xpress-FIP3) (subcloned from pEGFP-C1/FIP3 [26]); pcDNA3.1-HisB/FIP4 (Xpress-FIP4) (subcloned from pEGFP-C1/FIP4); pVJL10-TSG101 (PCR: template pEGFP-C1/TSG101, *Fwd* cccgaattcatggcggtgtcggagagccagc, *Rev* cccgaattcctagtagaggtcactgagaccgg); pVJL10-TSG101_1–234_ (SDM: *Fwd* gcctctctcatctctgcggtctaggacaaactgagatggcggatg, *Rev* catccgccatctcagtttgtcctagaccgcagagatgagagaggc); pVJL10-TSG101_235–313_ (PCR: *Fwd* cccgaattcagtgacaaactgagatggcggatga, *Rev* cccggatcctaatcattgttttcagactgattttccat); pVJL10-TSG101_235–257_ (SDM: *Fwd* gcagagctcaatgccttgaaatgaacagaagaagacctgaaaaag, *Rev* ctttttcaggtctttcttctgttcatttcaaggcattgagctctgc); pVJL10-TSG101_235–273_ (SDM: *Fwd* caccagaaactggaagagatgtgaacccgtttagatcaagaagta, *Rev* tacttcttgatctaaacgggttcacatctcttccagtttctggtg); pVJL10-TSG101_274–290_ (SDM: *Fwd* gttgataaaaacatagaactttgaaaaaagaaggatgaagaactc, *Rev* gagttcttcatccttcttttttcaaagttctatgtttttatcaac); pVJL10-TSG101_274–313_ (PCR: *Fwd* cccgaattcgttacccgtttagatcaagaag, *Rev* cccggatcctaatcattgttttcagactgattttccat); pVJL10-TSG101_235–390_ (PCR: *Fwd* cccgaattcagtgacaaactgagatggcggatga, *Rev* cccggatcctcagtagaggtcactgagaccg); pVJL10-TSG101 K257P (SDM: *Fwd* ggcagagctcaatgccttgccacgaacagaagaagacctg, *Rev* caggtcttcttctgttcgtggcaaggcattgagctctgcc); pVJL10-TSG101 V274P (SDM: *Fwd* accagaaactggaagagatgcctacccgtttagatcaagaag, *Rev* cttcttgatctaaacgggtaggcatctcttccagtttctggt); pVJL10-TSG101 N287P (SDM: *Fwd* tcaagaagtagccgaggttgataaacccatagaacttttgaaaaagaagga, *Rev* tccttctttttcaaaagttctatgggtttatcaacctcggctacttcttga); pLexA/Rab11a Q70L [42]; pGADGH (Clontech); pGADGH/RCP [42]; pGADGH/Rip11 [31]; pGADGH/FIP2 [31]; pGADGH/FIP3 (subcloned from pEGFP-C1/FIP3 [26]); pGADGH/FIP4 (subcloned from pEGFP-C1/FIP4); pGADGH/FIP4_2–363_ (subcloned from pEGFP-C1/FIP4_2–363_); pGADGH/FIP4_364–637_ (PCR: *Fwd* cccggatccactgaagagcaagctgaagcaagag, *Rev* cccgaattcttagtgtttgatctcgaggatggag); pGADGH/FIP4_364–519_ (PCR: *Fwd* cccggatccactgaagagcaagctgaagcaagag, *Rev* cccgaattctaccgctcgcagtccagcttgta); pGADGH/FIP4_364–412_ (PCR: *Fwd* cccggatccactgaagagcaagctgaagcaagag, *Rev* cccgaattctactccagcttgccgtaggcctcg);pGADGH/FIP4_413–474_ (PCR: *Fwd* cccggatccaagggagaaggctaccgaggtgg, *Rev* cccgaattctacaggtccatctcatctttgagcc); pGADGH/FIP4_475–519_ (PCR: *Fwd* cccggatccatacaagcgcatgatggacaagctg, *Rev* cccgaattctaccgctcgcagtccagcttgta); pGADGH/FIP4_540–571_ (PCR: *Fwd* cccggatccagtggagctcgagcacgaggtc, *Rev* cccgaattctagctgaggctcaaaatctgcccat); pGADGH/FIP4_572–637_ (PCR: *Fwd* cccggatccactctacgaagcaaaaaacctctttg, *Rev* cccgaattcttagtgtttgatctcgaggatggag); pGADGH/FIP4 L375P (subcloned from pEGFP-C1/FIP4 L375P); pGADGH/FIP4 E390P (SDM: *Fwd* tggtgaaggatcagccgaccacggccgagc, *Rev* gctcggccgtggtcggctgatccttcacca); pGADGH/FIP4 L443P (SDM: *Fwd* aacaacagtgactcggcccaagtctcaaacagaga, *Rev* tctctgtttgagacttgggccgagtcactgttgtt); pGADGH/FIP4 E453P (subcloned from pEGFP-C1/FIP4 E453P); pGADGH/FIP4 L487P (SDM: *Fwd* gcgacagaaccgccctgagttccagaagg, *Rev* ccttctggaactcagggcggttctgtcgc); pGADGH/FIP4 A495P (SDM: *Fwd* aggagcgggagccgacgcaggag, *Rev* ctcctgcgtcggctcccgctcct). All constructs generated by SDM or PCR were verified by DNA sequencing (Macrogen).

### Co-immunoprecipitation and immunoblotting

Subconfluent HeLa cells growing on 10 cm dishes were transfected with the 2 µg of each of the indicated plasmids. 16 hours post-transfection, the cells were lysed in 500 µl of lysis buffer (LB) [125 mM NaCl, 0.5% Igepal CA-630, 50 mM HEPES pH 7.4, 1 mM MgCl_2_, 1 mM AEBSF, plus complete Mini, EDTA-free protease inhibitor cocktail tablets (Roche)], passed twice through a 26-gauge needle, and incubated on ice for 10 min. Magnetic beads conjugated to sheep anti-mouse IgG (Dynal) were bound to mouse anti-Xpress antibodies. Antibody-coated beads were incubated with 440 µl of each of the lysates (60 µl was retained as starting material) for 3 h at 4°C, under rotation at 7 rpm. Antibody-coated bead/protein complexes were precipitated by placing the tubes on the dynabead magnet. Unbound proteins were removed, and the beads gently washed three times with 1 ml of LB. Specifically-associated proteins were eluted from the beads by boiling for 10 min in 60 µl of 1× Laemmli sample buffer. 60 µl of each of the lysates (starting material) was boiled in 30 µl of 3× Laemmli sample buffer. 25 µl of each of the starting materials and eluted samples were resolved by 12% SDS-PAGE and immunoblotted with anti-GFP and anti-Xpress antibodies. Immunoblotting analyses were performed on an Odyssey Infrared Imaging System and processed using the Odyssey Infrared Imaging Application Software (LI-COR), as described in [43]. Secondary antibodies used were IRDye 680 goat anti-rabbit and IRDye 800CW goat anti-mouse (LI-COR).

### Immunofluorescence, fluorescence microscopy and data analysis

Immunofluorescence microscopy was performed as previously described [44]. Secondary antibodies used were Alexa Fluor 594-conjugated goat anti-mouse (Molecular Probes) and Cy3 (indocarbocyanine)-conjugated donkey anti-rabbit (Jackson ImmunoResearch). Images were recorded in a temperature-controlled environment (18°C) using a Zeiss LSM 510 META confocal microscope fitted with a 63×1.4 plan apochromat lens. Images were processed using Zeiss LSM Image Browser or Zeiss ZEN Light Edition software and Adobe Illustrator. All micrographs shown are 3D projections from the optical sections of the entire Z-stack. To quantify cytokinesis failure in cells expressing GFP-fused proteins/polypeptides, a minimum of 150 transfected cells per experiment were counted and scored for multinucleation (>1 nucleus). Results, from three independent experiments, were expressed as the mean percentages ± S.D. Statistical significance was determined using the unpaired *t* test function of Excel (Microsoft), assuming one-tailed distributions and unequal variances. Significance differences were defined as discernable where p<0.05.

## Supporting Information

Figure S1 **Mutation of the TSG101 coiled-coil domain reduces the probability of coiled-coil formation.** Plots depicting the probability of α-helical coiled-coil structure formation in wild-type and mutant TSG101 as determined using the *PairCoil* algorithm.
(TIF)

Figure S2 **Mutation of the FIP4 coiled-coil domain reduces the probability of coiled-coil formation.** Plots depicting the probability of α-helical coiled-coil structure formation in wild-type and mutant FIP4 as determined using the *PairCoil* algorithm.
(TIF)

Figure S3 **Mutation of Rab11-binding domain of FIP4 perturbs its distribution.** (***A***) Portion of a *ClustalW* alignment of the FIPs. Identities are in black and similarities are in grey. The conserved Rab11-binding domain (RBD) is underlined in green and the conserved YID/YMD motif is underlined in blue. (***B***) HeLa cells were transfected with constructs encoding the indicated polypeptides. At 16–18 hours post-transfection, cells were processed for immunofluorescence microscopy and immunostained with an anti-Rab11a antibody. Images were acquired by confocal microscopy. Scale bar indicates 10 µm. Data are typical of at least three independent experiments.
(TIF)
