# Supplementary figures and images for: Tumor Susceptibility Gene 101 (TSG101) Is a Novel Binding-Partner for the Class II Rab11-FIPs
Source: PLoS One. 2012 Feb 14;7(2):e32030. doi: 10.1371/journal.pone.0032030 (PMC3279423; doi:10.1371/journal.pone.0032030)

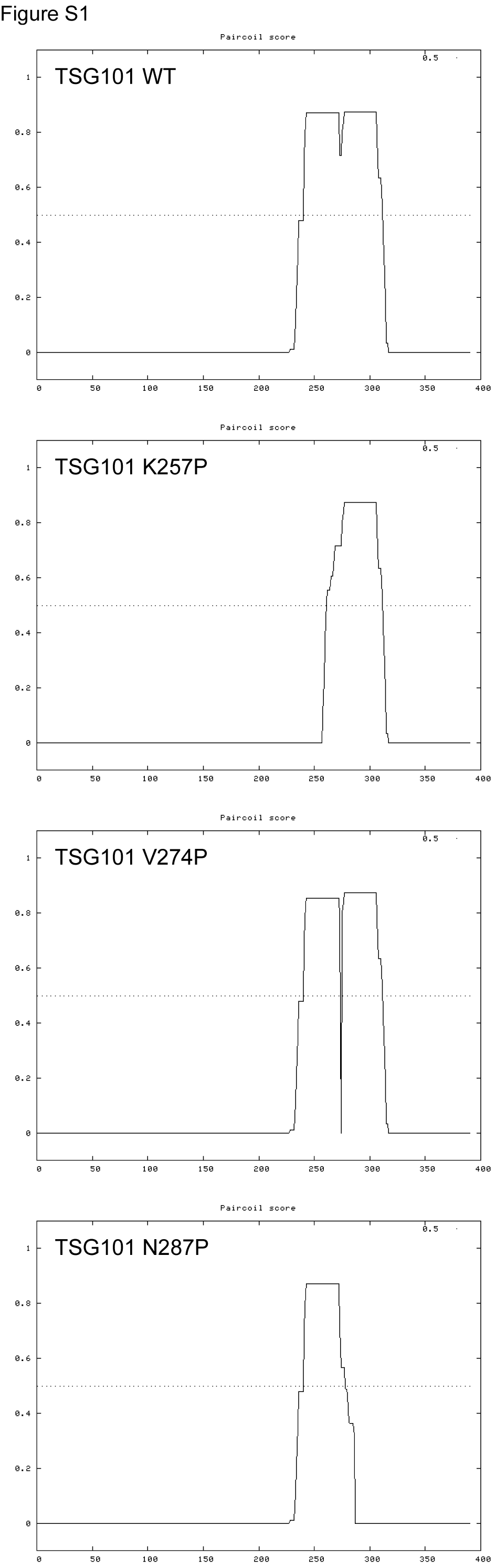

Supplement: Figure S1 — Mutation of the TSG101 coiled-coil domain reduces the probability of coiled-coil formation. Plots depicting the probability of α-helical coiled-coil structure formation in wild-type and mutant TSG101 as determined using the PairCoil algorithm. (TIF) [file pone.0032030.s001.tif]

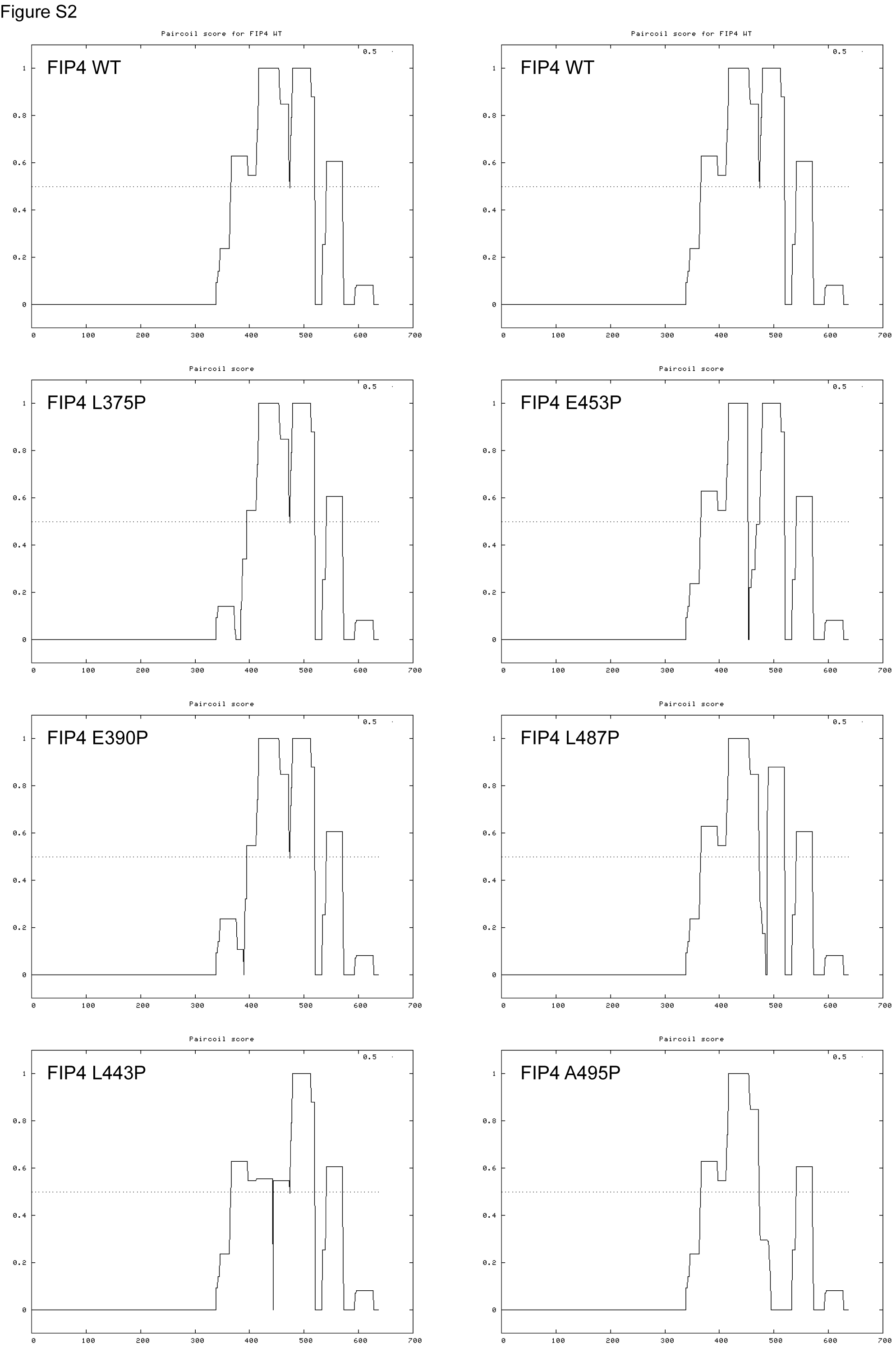

Supplement: Figure S2 — Mutation of the FIP4 coiled-coil domain reduces the probability of coiled-coil formation. Plots depicting the probability of α-helical coiled-coil structure formation in wild-type and mutant FIP4 as determined using the PairCoil algorithm. (TIF) [file pone.0032030.s002.tif]

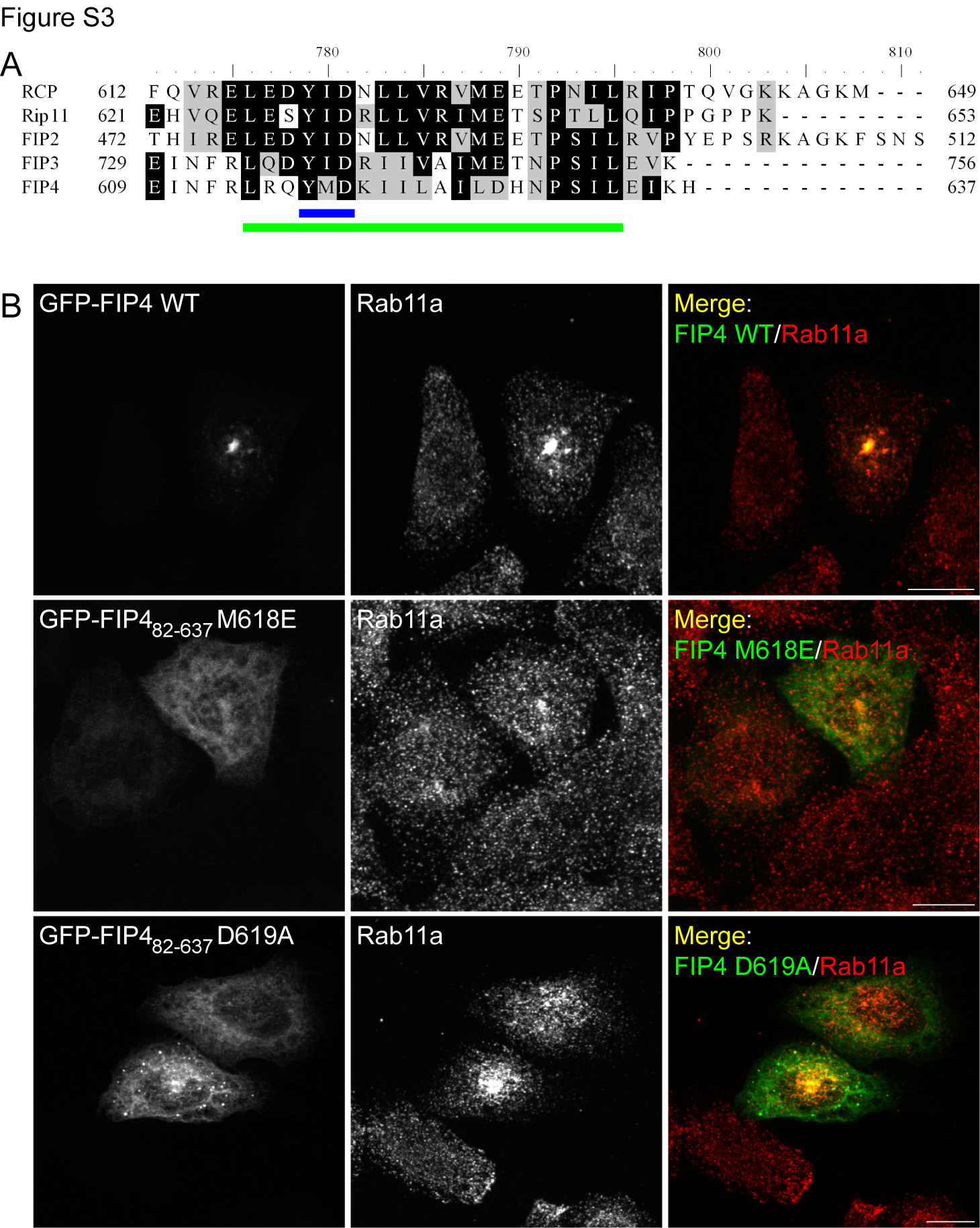

Supplement: Figure S3 — Mutation of Rab11-binding domain of FIP4 perturbs its distribution. (A) Portion of a ClustalW alignment of the FIPs. Identities are in black and similarities are in grey. The conserved Rab11-binding domain (RBD) is underlined in green and the conserved YID/YMD motif is underlined in blue. (B) HeLa cells were transfected with constructs encoding the indicated polypeptides. At 16–18 hours post-transfection, cells were processed for immunofluorescence microscopy and immunostained with an anti-Rab11a antibody. Images were acquired by confocal microscopy. Scale bar indicates 10 µm. Data are typical of at least three independent experiments. (TIF) [file pone.0032030.s003.tif]
